# Secure Patient Data Transfer Using Information Embedding and Hyperchaos

**DOI:** 10.3390/s21010282

**Published:** 2021-01-04

**Authors:** Hanan Aljuaid, Shabir A. Parah

**Affiliations:** 1 Department of Computer Science, College of Computer and Information Sciences, Princess Nourah Bint Abdulrahman University (PNU), Riyadh 84428, Saudi Arabia; haaljuaid@pnu.edu.sa; 2 Department of Electronics and IT, University of Kashmir, Srinagar 190006, India

**Keywords:** health 4.0, cyber-physical systems, reversible data hiding (RDH), security, reversibility, embedding

## Abstract

Health 4.0 is an extension of the Industry standard 4.0 which is aimed at the virtualization of health-care services. It employs core technologies and services for integrated management of electronic health records (EHRs), captured through various sensors. The EHR is processed and transmitted to distant experts for better diagnosis and improved healthcare delivery. However, for the successful implementation of Heath 4.0 many challenges do exist. One of the critical issues that needs attention is the security of EHRs in smart health systems. In this work, we have developed a new interpolation scheme capable of providing better quality cover media and supporting reversible EHR embedding. The scheme provides a double layer of security to the EHR by firstly using hyperchaos to encrypt the EHR. The encrypted EHR is reversibly embedded in the cover images produced by the proposed interpolation scheme. The proposed interpolation module has been found to provide better quality interpolated images. The proposed system provides an average peak signal to noise ratio (PSNR) of 52.38 dB for a high payload of 0.75 bits per pixel. In addition to embedding EHR, a fragile watermark (WM) is also encrypted using the hyperchaos embedded into the cover image for tamper detection and authentication of the received EHR. Experimental investigations reveal that our scheme provides improved performance for high contrast medical images (MI) when compared to various techniques for evaluation parameters like imperceptibility, reversibility, payload, and computational complexity. Given the attributes of the scheme, it can be used for enhancing the security of EHR in health 4.0.

## 1. Introduction

Technology development is changing the world scenario thick and fast. The multimedia and Internet are exponentially changing and revolutionizing all spheres of life. The present generation systems are smarter, self-organized, well-connected, interoperable, decentralized, and flexible. This has been made possible by adapting cyber-physical systems to monitor and control the machines while using the Internet of Things (IoT) for proper management of data and various system components [1,2,3]. It is aimed at progressive virtualization to enable the personalization of healthcare by catering to health care needs electronically. This is known as the Health 4.0 cyber-physical system (HCPS). Personalized healthcare delivery is being achieved by the efficient implementation of core technologies like artificial intelligence, cyber-physical systems, IoT-based architectures, and the evolution of 5G-based networked mobile communication [4,5,6]. The main design principles of Health 4.0 include virtualization, decentralization, real-time capability, modularity, and service orientation. It aims to fully replace conventional healthcare with electronic smart healthcare to allow personalized monitoring and diagnosis [7,8,9,10]. The Indian healthcare industry is growing exponentially and is expected to be at USD280 billion industry by 2020. Though to date, the healthcare industry is fragmented but certain super-specialty chains are moving towards Health 4.0. However, the recent developments like National Health Policy 2017, enhanced penetration of health insurance, aspirational middle-class, and the emergence of patient-centered care are paving the way for Health 4.0. The successful implementation and management of Health 4.0 include issues like data management, service interoperability, and security of personalized information among others. The protection of the sensitive information contained in the Electronic Health Record (EHR) can be identified as a major challenge for efficient implementation of the Health 4.0 initiative [11]. The virtualization of healthcare systems requires the wireless transmission of EHRs and other sensitive medical information among the healthcare providers and as such various security safeguards have to be put in place [12]. Many incidents of unauthorized data breaches have been reported globally as well as in the Indian healthcare industry which emphasizes the need for efficient security protocols. The severity of data breaches in India could be gauged from the fact that eighteen breaches have been reported in the first half of 2017, as per Gemalto’s breach level index [13,14]. This has led to 203.7 million data records being compromised [15]. The increased tendency of security breaches demands that security measures need to be improved for the successful implementation of Health 4.0 [16,17]. Though cryptography is being used as a major technology to ensure the security of EHRs, however, the camouflaged appearance of cryptographic data attracts more attention from the adversary and hence increases the chances of a security breach. The information (EHR) concealment in medical images has been presented as an efficient and alternate approach towards improving the security of patient data. It is seen that the integrated application of encryption along with steganography can generate highly secure and good quality Stego images (SI) [18]. An encryption-based cryptosystem along with the information hiding system must fulfill the requirements of security, generation of a uniform histogram, optimal value of entropy, payload, robustness, and imperceptibility [19,20]. For the data hiding system, a high level of imperceptibility leads to a reduced probability of an attack by an adversary. Further, if the hiding method used is reversible, the bandwidth required to exchange EHR in such a case is also less [21,22]. Of late, chaotic systems, due to their less computational complexity are being used for privacy preservation of e-health data. A few works describing the use of chaotic encryption for health-related data could be seen in [23,24].

This paper presents a dual-layer security system that uses hyperchaos based encryption and image interpolation (IP)-based EHR hiding scheme for medical images (MIs). The discussed IP scheme can provide better visual-quality cover images (CIs) in comparison to well-known interpolation schemes like INP, NMI, etc. To authenticate the EHR a fragile watermark (logo) is embedded in the CI using bit replacement. The use of hyperchaos results in a very large key-space making it highly resilient to brute force attacks. The proposed scheme uses a spatial domain approach for embedding and as such ensures lesser computational complexity to make the proposed security scheme well-suited for security scenarios like those required in Health 4.0.

The rest of the paper is organized as follows. Related work is described in Section 2. The proposed work has been described in detail in Section 3. The results obtained are presented in Section 4. A discussion of the results is presented in Section 5 and the paper concludes in Section 6.

## 2. Related Work

A lot of variants of data hiding (DH) techniques exist in the literature. However, for embedding in the patient diagnostic images, reversible data hiding (RDH) techniques are preferred. The reversibility of the CI can be accomplished through several techniques. Various, techniques based on data compression [25,26], difference expansion [27,28,29,30], and histogram bin shifting [31,32,33,34,35] have been adopted to achieve reversibility. The purpose behind using the compression methods [25,26] is to hide the characteristics of the secret data in the CI including the WM. Many RDH schemes using the difference expansion (DE) technique have also been utilized. A watermarking scheme illustrated by Tian et al. [27] uses the DE scheme. This method provides enhanced embedding capacity (EC) in addition to the reduced computational complexity compared to some existing techniques [28,29]. The histogram bin shifting based schemes have also been proposed where the lowest or the highest bin is shifted to embed bit ‘1’ or a bit ‘0’ [31,32,33,34,35].

The first IP-based reversible information embedding (IRIE) scheme was proposed by Jung and Yoo with improved capacity and image quality using the method of Neighbor-Mean-Interpolation (NMI) [36]. In [37], another reversible watermarking technique has been presented by Luo et al., where reversibility is accomplished using neighboring pixels. Their scheme properly manages the overflow and underflow conditions near the boundary. Abadi et al. [38] have developed a histogram profile shifting (HS) based method utilizing the pixels at the boundary to improve capacity. An MI-based RDH method has been proposed by Naheed et al. [39]. Lee and Huang [40] applied IP on neighboring pixels (INP) for RDH. Their reported technique improves upon the DH method developed by Jung and Yoo [36]. In their method, the maximum intensity is used to increase the contrast value calculated between adjacent values of the pixels. Furthermore, Lee et al. [41] also offered a scheme using a maximum difference calculated between adjacent pixels. Their reported scheme generates SI of reduced quality. Tang et al. [42] have presented another data embedding scheme with improved payload (PL) capability. Arsalan et al. [43] proposed a method based on the genetic algorithm (GA) and companding schemes that offer low information EC. A reversible secret DH technique for MI is demonstrated by Naheed et al. [44]. They create a balance in the embedding proportions and imperceptibility. However, it is seen that the use of PSO and GA renders high computational complexity with relatively low EC.

Wang et al. [45] describe a reversible HS-based DH technique. The IP error in the wall pixels is used to increase embedding capacity. Abdul Wahed et al. [46] presented an improved local dispersion-based IP scheme and analyzed the embedding performance for medical images. They also presented another IP scheme known as quadratic interpolation for RDH. Their scheme achieves a high EC with better SI-quality [47]. Chang et al. have proposed an RDH method for image IP based on the Sudoku matrix [48]. In this scheme, the distortion of SI is reduced by embedding secret bits in a pair of pixels. Their scheme also achieves a higher EC along with good quality for SI when compared to some of the previous research works. Mathew et al. presented an IP-based RDH for encrypted images [49]. Their illustrated scheme efficiently utilizes the reversible hiding schemes for unencrypted images to maximize the EC. The algorithm outperforms most of the contemporary DH approaches.

Robustness to various signal processing attacks is of vital importance, in addition to the security of embedded data in medical images. Some of the recent work with high robustness to various attacks could be seen in [50,51]. In [50] a method for the secure transmission of medical images has been presented. The scheme uses integer wavelet transform and chaotic sequences. The scheme, though robust, is computationally complex, as it has been implemented in the transform domain. Also, the scheme supports a very small payload of 0.18 bpp. A multilevel discrete wavelet transform and singular value decomposition-based scheme for medical has been reported in [51]. This scheme is highly robust for hiding watermarks, which could be patient information. The transform domain implementation of this scheme makes it computationally complex. The security of the embedded data has been ensured utilizing chaos theory in both cases, as chaotic maps are very efficient for image security [52].

In this work, we have presented an efficient scheme that can hide the encrypted EHR in an interpolated CI developed from a seed image. The proposed IP algorithm generates a better perceptual model of a CI from seed image compared to many contemporary schemes. We use the generated CI for embedding EHR information. For encryption of the EHR hyperchaos (HC) based technique is employed because of the advantages like ergodicity, a high value of randomness, very intense dependence on initial conditions and parameters [53]. HC systems generate strong encryption algorithms because of their highly aperiodic behavior. The chaos-based ciphers provide robust confusion and diffusion of the input data by the process of pixel transposition and value substitution.

Pertinent to mention, though 1-D chaos-based algorithms are simple to implement but have disadvantages like less robustness, simpler dynamical nature, and reduced keyspace [54,55,56,57,58,59,60,61,62,63,64]. On the other hand, the HC systems have complex dynamical behavior, better key sensitivity, and larger keyspace. Hyperchaotic systems have two positive Lyapunov exponents and are therefore highly unpredictable. These strengths offered by the HC system have led to the development of many improved algorithms [65].

Kun Zhan et al. have illustrated an HC-based system for image cryptography [66]. Their proposed scheme aims to apply the pseudo-random sequence (PRN) sequence developed by the HC system to the underlying processes of the encryption algorithm. In this paper, we also apply the 4-D HC system to scramble the WM logo embedded to authenticate the transferred content and the EHR of the patient. As the HC system has high key sensitivity and any slight change in the initial conditions will lead to a different sequence being generated, which provides high security to the EHR data.

## 3. Proposed Technique

The presented reversible EHR embedding based on an HC encryption and IP algorithm has been discussed in this section. An image IP algorithm is used to achieve the reversibility of the actual up-sampled image. This technique can be efficiently used to construct a CI from the original (ORG) clinical image that can be represented by Equations (1)–(4). The proposed technique is illustrated in the following sub-sections. Section 3.1 describes the detailed process of the IP technique for CI formation. The IP technique can provide high embedding capacity along with the generation of better visual quality. Section 3.2 describes in detail Chen’s HC system employed to generate the PRN sequence. A PRN sequence is generated and is utilized to encrypt the EHR data and the authentication logo. Both the EHR and the fragile WM (logo) are implanted into the interpolated CI. The EHR and WM bits are embedded with the help of the spatial domain algorithm called the least significant bit (LSB) substitution technique. Further, Section 3.3 and Section 3.4 respectively deal with the process of data embedding and data reconstruction.

### 3.1. Improved IP Scheme

**Figure 1:**
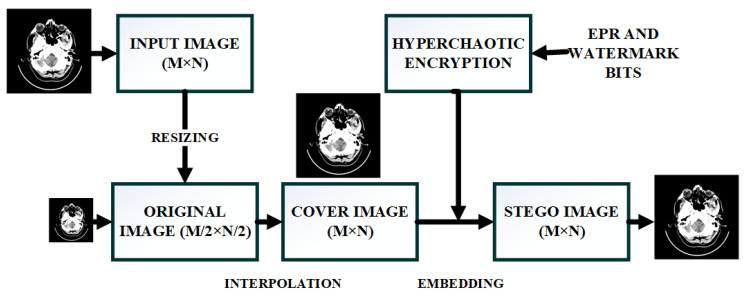
A detailed sequence of operations for the presented method.

The detailed sequence of operations for the presented method with all the underlying operations is shown in Figure 1.

The CI generation involves down-sizing followed by the subsequent application of IP. Firstly, the inputted image (O) of M × N dimensions has been decreased in size to form the original (ORG) image (I) of dimensions equal to M/2 × N/2 with the help of Bilinear Transformation (BT)-based technique. Then IP is utilized to scale-up the ORG image (I) into the CI-(*I*1). The process of *I*1 generation from the original image is illustrated in the subsequent steps:

- One-pixel *I*1(*k*, *l*) of the CI is equivalent to *I*(*k*, *l*) of the ORG image (I).
- We calculate the pixel values *I*1(*k*, *l* + 1), *I*1 (*k* + 1, *l*), and *I*1 (*k* + 1, *l* + 1) by making use of Equations (1)–(4).

The following equations can be utilized to scale up the image:

(1) I1(k,l)=I(k,l),

(2) $$I1\left(k+1,l\right)=\frac{\sqrt{I\left(k,l\right)\times I\left(k+1,l\right)}+I\left(k,l\right)\;}{2},$$

(3) $$I1\left(k,l+1\right)=\frac{\sqrt{I\left(k,l\right)\times I\left(k,l+1\right)}+I\left(k,l\right)}{2},$$

(4) $$I1\left(k+1,l+1\right)=\sqrt{I1\left(k+1,l\right)\times I1\left(k,l+1\right)},$$

**Figure 2:**
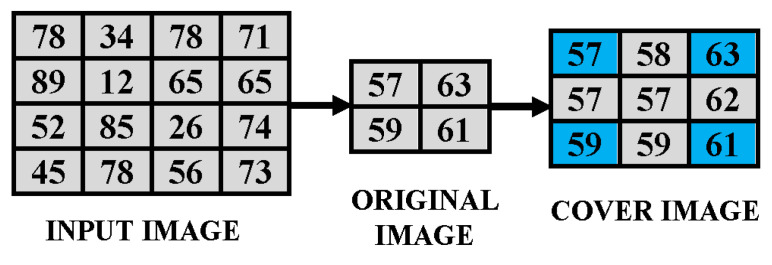
Explanation of the process of IP or scaling up of Image.

The pixel *I*1(*k*, l) is the seed pixel also known as the seed/pivot pixel. With the help of the above-mentioned equations, all the non-seed pixels of the interpolated CI can be generated. The CI has very good quality as compared to the input image (O) which is indicated by the high PSNR value between the two. Figure 2 explains the method by which the resized image is up-sampled to form the CI for embedding.

### 3.2. Hypechaotic (HC) Encryption

Hyperchaos arises when a high-dimensional non-linear system has more than two positive Lyapunov exponents. HC systems have very complex dynamical behavior and are highly secure and unpredictable. They depend upon many parameters and have a greater number of initial conditions, which increases the key-space of the encryption algorithm. In this paper, we employ the 4-D Chen’s HC system to strengthen the protection of the EHR data and WM bits embedded inside the MI. Chen’s 4-D HC system has a set of four non-linear equations indicated in equation set (5) which governs its behavior. The system is in HC state for the value of control parameters as *a* = 35, *b* = 3, *e* = 35, *t* = 5, $l_{1}=\;1$, $l_{2}=\;0.2,l_{3}=\;0.3$. The initial conditions are $s_{1}\left(1,\;1\right)\;=\;0.12,$ $s_{2}\left(1,\;1\right)\;=\;0.23,$ $s_{3}\left(1,\;1\right)\;=\;0.34,$ and $s_{4}\left(1,\;1\right)\;=\;0.45$ [66]. The HC sequence has to be pre-iterated several times to remove the initial adverse effects and the value of time set ‘h’ is taken as 0.001.

(5) $$\dot{s_{1}}=\;a\left(s_{2}-\;s_{1}\right)+\;l_{1}s_{4},\;\dot{s_{2}}=\;es_{1}-\;s_{1}s_{3}+\;l_{2}s_{4},\;\dot{s_{3}}\;=\;-bs_{3}-\;s_{1}s_{2}+\;l_{3}s_{4,}\;\dot{s_{4}}=-\;ts_{1},$$

For the HC system, the state variables $x_{1}^{j}$, $x_{2}^{j}$, $x_{3}^{j}$ and $x_{4}^{j}$ are calculated for the M × N iterations of the system and two key sequences ${\left(S_{i}^{a}\right)}^{j}$ and ${\left(S_{i}^{b}\right)}^{j}$ will be formed, where ‘*j*’ represents the index of operation. ${\left(S_{i}^{a}\right)}^{j}$ and ${\left(S_{i}^{b}\right)}^{j}$ values are concatenated to develop a sequence $S^{j}$ which is represented by Equation (6), where *i* = 1, 2, 3, 4 and ${\left(S_{i}^{a}\right)}^{j},\;{\left(S_{i}^{b}\right)}^{j}\epsilon \;\left[0,255\right].$

(6) $$S^{j}\;=\;[{\left(S_{1}^{a}\right)}^{j},\;{\left(S_{2}^{a}\right)}^{j},\;{\left(S_{3}^{a}\right)}^{j},\;{\left(S_{4}^{a}\right)}^{j},\;{\left(S_{1}^{b}\right)}^{j},\;{\left(S_{2}^{b}\right)}^{j},\;{\left(S_{3}^{b}\right)}^{j},\;{\left(S_{4}^{b}\right)}^{j}],\;$$

The PRN sequence ‘*k*’ is formed by joining the $S^{j}$ sequences as shown in Equation (7):

(7) $$k\;=\;\left[S^{1},\;S^{2},S^{3},\ldots ,S^{m\times n}\right]$$

The values of the sequence ‘*k*’ are XORed with the binary watermark and the EHR data which is to be embedded. The receiver needs to provide the same set of input keys which include the initial values and control parameters of the HC system to recover the authentication logo and the encrypted EHR data.

### 3.3. Data Embedding

We illustrate the method of implanting the Electronic Health Record (EHR) and the WM. As an initial step, the cover image pixels are distinguished as either seed pixels (SP) and non-seed pixels or data containing pixels (DCP). To assist in the proper reversibility only the non-seed pixels have been utilized for encrypted EHR embedding and no embedding is performed using seed pixels. Furthermore, an encrypted fragile WM has also been put in the cover media. The data vector that is thus implanted in the CI comprises of EHR bits and watermark bits (WM) and has been obtained by concatenating the individual vectors as:

Data vector = [WM: EHR]

The embedding of a fragile watermark helps detect any possible interference with the EHR. The proposed method is described as Algorithm 1.
**Algorithm 1.** Image IP, EHR, and WM Encryption using HC, and LSB embedding of data bits in DCP**Input:** Grayscale M × N Secret Image, a hyperchaotic map with the initial values s1, s2, s3, s4′ and control parameters a, b, e, t, l1, l2, l3.**Output:** Stego Image (SI) of size M × N  **BEGIN**s1, s2, s3, s4 ← initial condition for hyperchaosa, b, e, t, l1, l2, l3 ← control parameters for hyperchaos**for** n = 2: x^2^$\dot{s_{1\left(n+1\right)}}$= $a\left(s_{2}-\;s_{1}\right)+\;l_{1}s_{4}$$\dot{s_{2\left(n+1\right)}}=\;es_{1}-\;s_{1}s_{3}+\;l_{2}s_{4}$$\dot{s_{3\left(n+1\right)}}$ = −b$s_{3}-\;s_{1}s_{2}+\;l_{3}s_{4,}$$\dot{s_{4\left(n+1\right)}}=-\;ts_{1}$**end for**Calculate $x_{1}^{j}$, $x_{2}^{j}$, $x_{3}^{j}$ and $x_{4}^{j}$ as state variables to perform key sequences ${\left(S_{i}^{a}\right)}^{j}$ and ${\left(S_{i}^{b}\right)}^{j}$concatenate the values as $S^{j}$$S^{j}\;=\;[{\left(S_{1}^{a}\right)}^{j},\;{\left(S_{2}^{a}\right)}^{j},\;{\left(S_{3}^{a}\right)}^{j},\;{\left(S_{4}^{a}\right)}^{j},\;{\left(S_{1}^{b}\right)}^{j},\;{\left(S_{2}^{b}\right)}^{j},\;{\left(S_{3}^{b}\right)}^{j}$, ${\left(S_{4}^{b}\right)}^{j}$],Obtain PRN sequence as$k\;=\;\left[S^{1},\;S^{2},S^{3},\ldots ,S^{m\times n}\right]$resize the input image**for rounds** ←   1: x   **for rounds**  ← 1: yInterpolate using Equations (1)–(4)**end for****end for**Data vector=[WM: EHR]   **for rounds**   ←   1: length (Data vector)Data=Data vector ⊕ k**end**Start embedding data in LSB’s of DCP pixels of the cover image by encrypted data vector (Data)**END**

### 3.4. Data Extraction

In this section, we illustrate the reverse function of the embedding process. The secret bits are extricated from the LSB of the non-seed pixels of the SI. The bits extricated from the cover media include the EHR and the WM. The drawn-out bits are distinguished into WM and EHR content. Subsequently, decryption is performed by the XOR operation between the encrypted WM bits and the HC-PRN sequence to generate the WM logo. The WM bits thus received are reshaped into a logo of size 128 × 128. If it is observed that the WM is affected in any manner, the EHR is not extracted and a request for retransmission is initiated. However, if the WM is correctly decrypted, then the EHR data bits are extracted and decrypted using the HC-PRN sequence. The seed pixels from each block are acquired to reconstruct the original image. The data extraction method is described as Algorithm 2.
**Algorithm 2.** CI generation, WM, and EHR data extraction and decryption**Input:** Stego Image (SI) M × N, keys as initial conditions and control variables **Output:** Cover Image (CI) of size M × N, WM, EHR  **BEGIN**s1′, s2′, s3′, s4′ ← key as an initial condition for hyperchaosa′, b′, e′, t’, l1′, l2′, l3′ ← control parameters for hyperchaos**for** n1 = 2: x1^2^$\dot{s\prime_{1\;\left(n1+1\right)}}$ = $a\prime \left(s_{2}\prime -\;s_{1}\prime \right)+\;l_{1}\prime s_{4}\prime$$\dot{s\prime_{2\left(n1+1\right)}}=\;e\prime s_{1}\prime -\;s_{1}\prime s_{3}\prime +\;l_{2}\prime s_{4}\prime$$\dot{s\prime_{3\left(n1+1\right)}}$ = −b$\prime s_{3}\prime -\;s_{1}\prime s_{2}\prime +\;l_{3}\prime s_{4}\prime$$\dot{s\prime_{4\left(n1+1\right)}}=-\;t\prime s_{1}\prime$**end for**Obtain PRN sequenceExtricate the LSB bits from the non-seed pixels of the SI to form the encrypted WM and encrypted EHR data bit vectors    **for rounds**  ←  1: length (extracted data vector)Original data vector = extracted data vector ⊕ k**End**Obtain the WM and EHR from this data vectorReshape the original WM to check if it has tamperedReconstruct the CI using the seed pixels.**END**

## 4. Experimental Results

**Figure 3:**
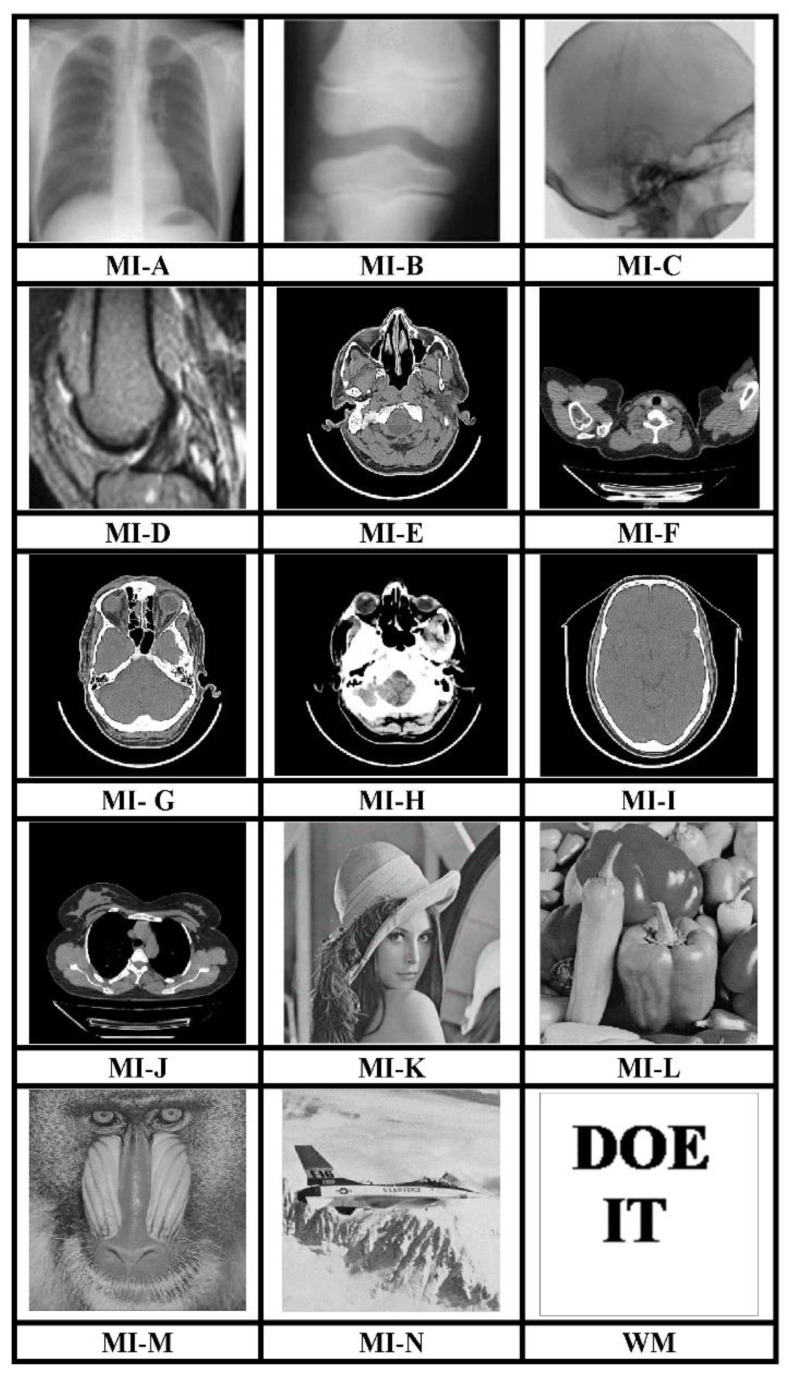
512 × 512 test images and 128 × 128 binary WM.

The MATLAB R2017a platform has been utilized for carrying out the experimental investigations for different gray-scale natural and MI. Both types of images that have been used for testing have dimensions 512 × 512. We have conducted experiments using a 64-bit Windows 10 Operating system with an i5 processor, 8 GB RAM, and 2.40 GHz clock speed. The different images, as well as a logo as WM, have been represented in Figure 3. The binary WM employed for authentication purposes has a 128 × 128 size. The technique is evaluated for a payload of 196,608 bits or 0.75 bits per pixel (bpp). The scheme reports an average encryption speed close to 1Mbps. The image quality metrics applied for evaluation of the scheme include normalized cross-correlation (NCC), peak signal to noise Ratio (PSNR), and structural similarity measure index (SSIM) [16,17,18,19,20]. In-depth analysis has been performed, which includes imperceptibility analysis, computational complexity analysis, and fragility analysis. Furthermore, a detailed comparison of many contemporary techniques has been described. Furthermore, the fragility analysis carried out reveals that the WM is fragile to all possible attacks and can easily detect tampering of data.

### 4.1. Imperceptibility Analysis

**Figure 4:**
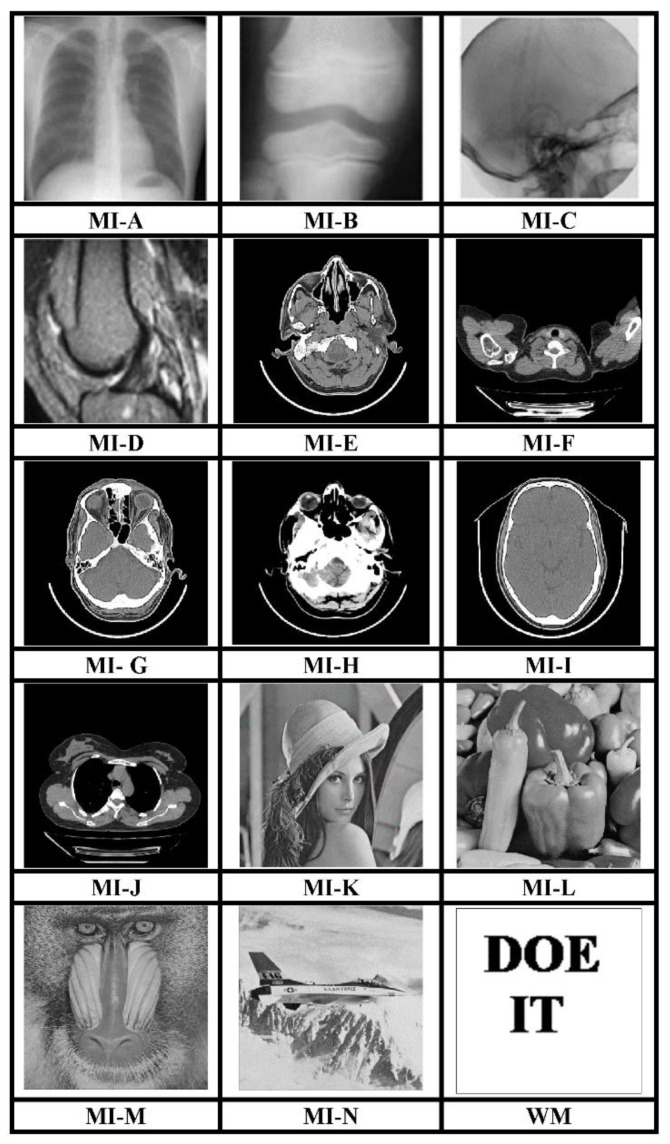
CI (Interpolated).

The main attribute of the presented image interpolation method is its capability of generating perceptually better-quality CI. The CIs generated by this scheme are of very high quality as indicated in Figure 4 and the evaluation quality metrics which include the PSNR1 and SSIM1 have been presented in Table 1. The scheme performs equally well for the natural as well as the MI.

### 4.2. Computational Complexity Analysis

The analysis of computation complexity for CI generation time is illustrated for various test images in Table 2. A detailed comparison of the CI generation time of our technique with two important interpolation schemes NMI [36] and INP [40] is presented below.

It could be seen that despite providing better quality CI, our scheme does not require any extra computational effort. The proposed interpolation method takes lesser time compared to the state-of-art-NMI and INP schemes as is evident. This makes the developed system a potential candidate for real-time imaging systems.

### 4.3. Payload and Reversibility Analysis

**Figure 5:**
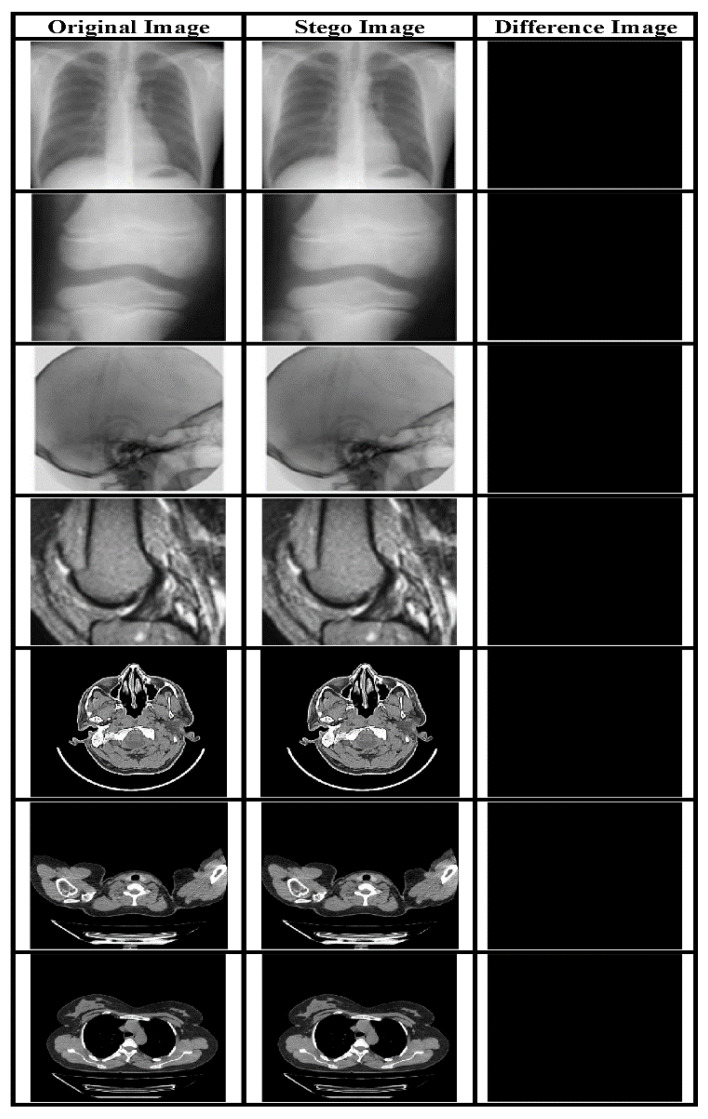
MI with their corresponding SI for a payload of 0.75 bpp and the subtracted image.

**Figure 6:**
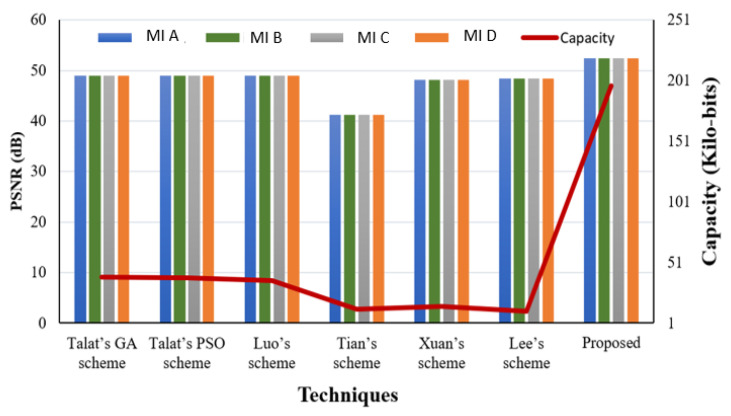
Perceptual transparency comparison.

**Figure 7:**
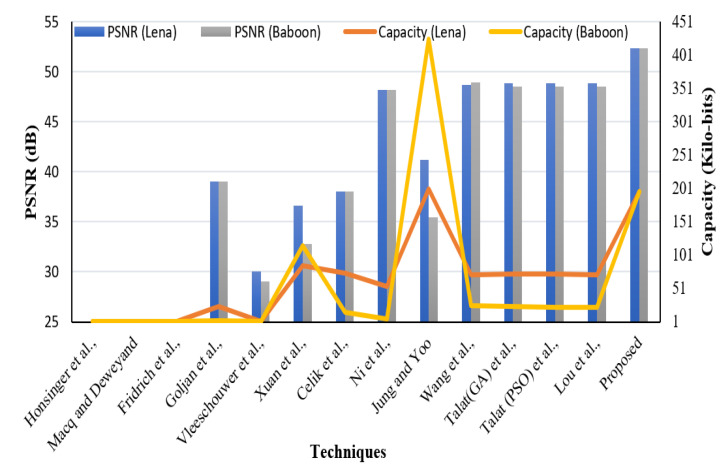
Perceptual transparency comparison.

The payload is a critical underlying requirement of a data hiding system. The perceptual quality of the SI is evaluated using subjective and quality metrics like PSNR and it is a direct function of payload. In our proposed system we have embedded two types of data in the CI obtained using a novel IP method: EHR and watermark. Both types have been embedded using the LSB approach. The visual quality of the SI yielded by the embedding method is evaluated based on objective as well as subjective analysis. Figure 5 presents various original and Stego-MIs with a payload value equal to 0.75 bpp. The generated SI’s quality is much superior, along with high payload value. Furthermore, to validate the described RDH method a comparison has been made with contemporary works mentioned in [44] as shown in Figure 6 and Figure 7.

The value of avg. PSNR for MI is 52.3756 dB whereas the avg. SSIM is 0.9849. This indicates that the scheme can yield high-quality images with a considerably high payload.

The PSNR2 and SSIM2 value for SI and the said payload is illustrated in Table 3.

Table 3 results depict that our system is capable of providing high visual quality images despite embedding an average payload of 0.75 bpp reversibly. This data embedding capacity is more than four times compared to 0.18 bpp [24]. Also, our scheme being implemented in the spatial domain is computationally less complex compared to [24].

In the comparison of the schemes for EC and PSNR-evaluation parameters, it is evident that our scheme performs better than many contemporary methods. The PSNR is comparable and the security offered to the WM and EHR is improved.

### 4.4. Fragility Analysis for Noise Attacks, Filtering Attacks, and Compression Attacks

The various watermarking images obtained have been attacked using different attacks as depicted in Table 4. This has been done to evaluate the fragility analysis of the proposed scheme.

Table 4 shows the salt and pepper noise with noise density 0.01 can produce recognizable WM. The effect of other attacks like the Gaussian noise (0.0001), Median Filtering (MF) attacks, and JPEG 50 compression are also shown in Table 4. It is concluded that the fragile WM can detect many types of tampering attempts like noise attack, filtering attack, and compression attacks of various quality factors among many others. Table 5 represents the value of NCC for different attacked watermarks.

The NCC values presented in Table 5 for various attacks reflect that our system is capable of detecting any attack. This is evident that for even a low strength attack, we are unable to recover the authentication WM.

### 4.5. Comparison of Proposed Scheme with Contemporary Methods

We have compared our scheme with several state of art techniques. The comparison of our scheme in terms of payload, BPP, and SSIM values are shown in Table 6 with reported works in [25,27,37,39,40,44]. The results depicted in Table 6 show that the proposed scheme provides high-quality SIs despite high embedding payload.

Furthermore, a comparison of the scheme has been made with [24,25,26,28,33,34,36,37,39,44,55] for PSNR and payload for natural images MI-K and MI-M as shown in Table 7.

Table 6 and Table 7 that our scheme provides better quality SIs, irrespective of the fact whether the cover media is a medical image or a natural image. In addition to this, unlike most of the schemes under comparison, like [24,25,26,33,34,37,39,45], our scheme makes use of a fragile WM for ensuring the early tamper detection at the receiver. This feature coupled with reversibility makes the proposed scheme a good candidate for securing patient records in e-health.

### 4.6. Key-Space and Statistical Analysis

The proposed technique utilizes Chen’s hyperchaotic system which utilizes four initial values s1, s2, s3, s4, and seven control parameters a, b, e, t, l1, l2, l3. Thus a total number of eleven parameters need to be set to define the actual keyspace. We have chosen the precision levels of all the eleven quantities as $10^{-10}$. This results in a total key space of $10^{110\;}$(~2^360^) which is extremely large and as such the proposed scheme is robust to the brute-force attack. A comparison of Key space with state of the art has been shown in Table 8. It is evident that our scheme provides better key space compared to state-of-the-art. Additionally, statistical analysis is of vital importance to determine strength of encrypted data. The effectiveness of the hyperchaotic system used in this work has been reported in [67,68] by successfully subjecting it to various tests of Statistical Test suite (NIST SP 800-22).

### 4.7. Encryption Speed

Speed of data encryption is an important parameter. Chen’s 4-D HC system used for chaotic sequence generation has a set of four non-linear equations that use eleven control parameters. The overall speed of the encryption depends on computational time of chaotic sequence followed by its use for data encryption. Table 9 provides the speed analysis of proposed system compared to state of art. As observed, our scheme outperforms those under comparison.

### 4.8. Histogram Analysis

**Figure 8:**
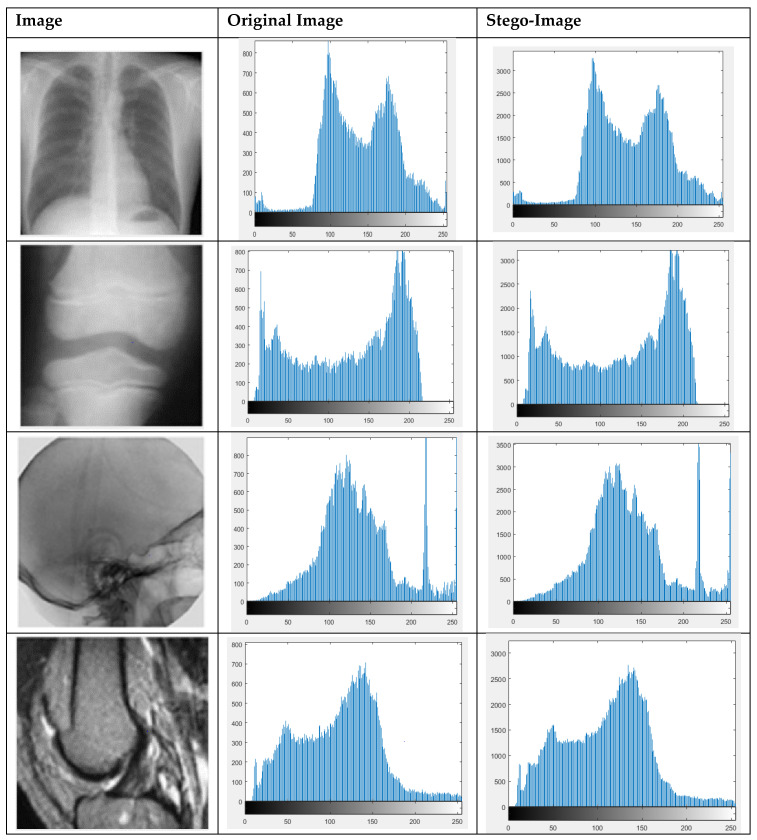
Histogram Analysis.

When data is embedded in a cover image, it changes its various parameters. One of the important requirements of a data embedding system that keeps statistical attacks at bay is that stego-image histogram should not significantly alter when data are embedded in it. Ideally, the cover and stego-image histograms should be identical. The more the two histograms are identical, the less information a histogram of the stego- image reveals about data embedded into it. We have tested our scheme for histogram analysis at a payload of 0.75 bpp. The results for some of the images have been depicted in Figure 8. The histograms of the original and stego-images are highly identical, indicating that the proposed scheme is less susceptible to statistical attacks.

## 5. Discussion

In this section, we present a discussion regarding the capability of the proposed method for its application in Health 4.0. The main aim of this work is to develop a scheme having the ability to enhance the security of EHR. In a smart-health system, while transferring EHR to the receiver, many parameters are of significant importance. These include the security of EHR, lossless nature of diagnostic data, authentication of the received content, and less computational complexity involved.

Towards this end, we have developed a dual-layer security framework for EHR utilizing a hyperchaotic algorithm and a newly developed interpolation scheme. The developed interpolation scheme ensures reversibility, hence avoids any loss of cover media information, facilitating better diagnosis. It was observed that our interpolation scheme performs better on both the fronts of the visual quality of stego-images and computational complexity. This has been observed by evaluating various parameters including imperceptibility, computational complexity, reversibility, and fragility.

The stego-module used to embed EHR has been evaluated using various objective parameters, which shows that the scheme provides an average PSNR of more than 52 dB for a payload of 0.75 bpp while maintaining reversibility. A comparison with state-of-art techniques reveals that the scheme is efficient enough to handle a higher payload while maintaining imperceptivity. For an embedding rate of 196,608 bits, an average PSNR of 52.3866 dB has been obtained for this method which is higher by 3.37 dB from the scheme in [39,44], 11.19 dB higher than the scheme present in [27]. The keyspace analysis shows that our system is highly robust to brute force attacks, while as histogram analysis also reveals that the proposed scheme is robust to statistical attacks.

The use of hyperchaotic encryption and the fact that data has been embedded in the spatial domain result in relatively lesser computational complexity compared to transform domain embedding. The watermark embedded within the image has been used to check the tampered regions (if any) at the receiving end. Fragility analysis shows that the proposed scheme can detect the presence of the tampers for various possible attacks which is a very important parameter while designing an e-health data security scheme.

## 6. Conclusions

Security of the EHR and authentication of the received content is significantly important in e-health services. This paper presents a dual-layer security mechanism for EHR security. We make use of steganography and cryptography to develop an efficient and secure system. Firstly a new interpolation method is developed and analyzed. It has been shown that the proposed interpolation method is capable of producing better CIs compared to some well-known methods like INP and NMI. The data to be embedded in the CIs generated by the proposed interpolation scheme is encrypted using a hyperchaotic system. This results in a highly secure system as a result of a very high keyspace of 10^110^. The embedded data includes EHR and a 128 × 128 authentication logo. To ensure reversibility of the cover images, no embedding is done at seed pixels, leaving them un-altered. The proposed scheme provides an average PSNR of more than 52 dB for a payload of 0.75 bpp. A comparison with various state-of-art techniques shows that our scheme is efficient enough to handle a higher payload while maintaining imperceptivity and reversibility. We have carried out the authentication analysis of our scheme to various signal processing attacks, and it shows that it can successfully detect various alterations. Thus the authenticity of the received content could be verified utilizing the proposed scheme. Also, the histogram analysis shows that it provides stego-images with a closely matched histogram to that of CIs. This minimizes any chances of statistical attacks. The scheme has been implemented in the spatial domain and, as such, is computationally efficient. Given all the features of the proposed scheme, it can be utilized for the security and authentication of EHR in Health 4.0. In the future, we aim to improve the data hiding capacity and computational complexity of our scheme by utilizing new chaotic maps.

## Figures and Tables

**Table 1:** Quality Objective Metrics.

MI	PSNR1 (dB)	SSIM1
MI-A	37.4057	0.9683
MI-B	45.2522	0.9913
MI-C	42.3601	0.9890
MI-D	41.4221	0.9831
MI-E	22.2075	0.8986
MI-F	26.5480	0.9114
MI-G	21.7612	0.8765
MI-H	25.0632	0.9560
MI-I	21.6530	0.8302
MI-K	32.9281	0.9479
MI-L	30.4135	0.8690
MI-M	24.4164	0.6996
MI-N	29.2605	0.9162
MI-J	27.5035	0.9371

**Table 2:** Comparison of CI Generation Time in seconds.

Images	NM1 [36]	INP [40]	Proposed
MI A	0.0781	0.0625	0.0625
MI B	0.0625	0.0781	0.0625
MI C	0.625	0.0938	0.0625
MI D	0.0781	0.0983	0.0625
MI E	0.0938	0.0625	0.0625
MI F	0.0938	0.0625	0.0625
MI G	0.0781	0.0625	0.0625
MI H	0.0938	0.0625	0.0625
MI I	0.0938	0.0625	0.0625
MI J	0.0781	0.0625	0.0625
MI-K	0.0781	0.0625	0.0625
MI-M	0.0938	0.0625	0.0625
MI-L	0.0781	0.0625	0.0625
MI-N	0.0781	0.0938	0.0781

**Table 3:** Objective quality metrics obtained using the proposed method.

Images	PSNR2 (dB)	SSIM2
MI A	52.4042	0.9953
MI B	52.3788	0.9948
MI C	52.3882	0.9953
MI D	52.3928	0.9967
MI E	52.3911	0.9836
MI F	52.1761	0.9002
MI G	52.3696	0.9844
MI H	52.3920	0.9827
MI I	52.3967	0.9858
MI J	52.3902	0.9833
MI-K	52.4063	0.9967
MI-M	52.3835	0.9964
MI-L	52.4021	0.9980
MI-N	52.3875	0.9964
Average	52.3756	0.9849

**Table 4:** Subjective quality images after various attacks.

Effect of Salt and Pepper Noise on the Extracted Watermark for Authentication				Effect of Gaussian Noise (0.001) on the Extracted Watermark for Authentication		
Attacked Image	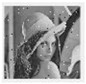	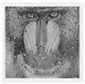	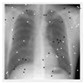	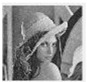	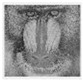	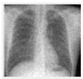
Extracted Logo	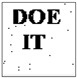	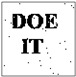	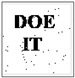	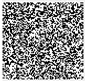	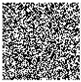	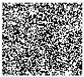
Attacked Image	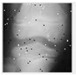	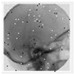	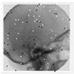	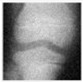	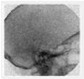	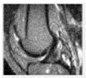
Extracted Logo	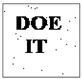	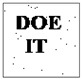	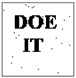	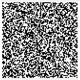	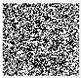	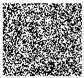
**Effect of Median Filtering on the extracted Watermark for Authentication**				**Effect of jpeg 50 compression on the extracted Watermark for Authentication**		
Attacked Image	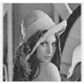	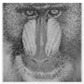	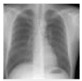	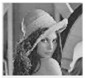	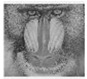	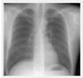
Extracted Logo	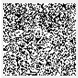	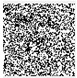	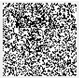	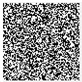	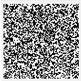	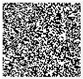
Attacked Image	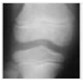	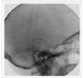	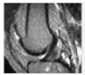	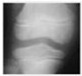	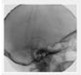	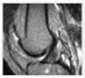
Extracted Logo	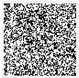	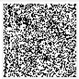	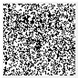	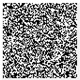	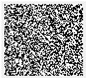	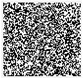

**Table 5:** NCC for various attacks.

Stego-Images	Salt and Peppers (0.01)	Gaussian Noise (0.0001)	MF	JPEG 50
MI-A	0.9939	0.5022	0.6018	0.4970
MI-B	0.9952	0.4975	0.5911	0.4995
MI-C	0.9943	0.5052	0.5870	0.5005
MI-D	0.9952	0.5032	0.6221	0.5024
MI-K	0.9960	0.5094	0.5957	0.5059
MI-M	0.9952	0.4930	0.5980	0.5092

**Table 6:** Comparison in terms of quality metric.

Image	Methods	EC (bits)	PSNR (dB)	BPP	SSIM
MI-A	[25]	14,614	48.1437	0.1282	0.9980
	[27]	12,217	41.1985	0.1082	0.9905
	[37]	36,060	48.9464	0.3194	0.9985
	[39]	38,700	49.0119	0.3427	0.9985
	[40]	10,882	48.4208	0.0963	0.9988
	[44]	38,390	49.0047	0.3400	0.9985
	Proposed	196,608	52.3866	0.75	0.9951
MI-B	[25]	14,614	48.1437	0.1282	0.9980
	[27]	12,217	41.1985	0.1082	0.9905
	[37]	36,060	48.9464	0.3194	0.9985
	[39]	38,700	49.0119	0.3427	0.9985
	[40]	10,882	48.4208	0.0963	0.9988
	[44]	38,390	49.0047	0.3400	0.9985
	Proposed	196,608	52.3859	0.75	0.9949
MI-C	[25]	14,614	48.1437	0.1282	0.9980
	[27]	12,217	41.1985	0.1082	0.990596
	[37]	36,060	48.9464	0.3194	0.9985
	[40]	10,882	48.4208	0.0963	0.9988
	[39]	38,700	49.0119	0.3427	0.9985
	[44]	38,390	49.0047	0.3400	0.9985
	Proposed	196,608	52.3865	0.75	0.9953
MI-D	[25]	14,614	48.1437	0.1282	0.9980
	[27]	12,217	41.1985	0.1082	0.9905
	[37]	36,060	48.9464	0.3194	0.9985
	[39]	38,700	49.0119	0.3427	0.9985
	[40]	10,882	48.4208	0.0963	0.9988
	[44]	38,390	49.0047	0.3400	0.9985
	Proposed	196,608	52.3857	0.75	0.9968

**Table 7:** Comparison of the proposed scheme for Lena and baboon images.

Technique	MI-K		MI-M	
	EC (bits)	PSNR (dB)	EC (bits)	PSNR (dB)
[24]	24,108	39.0	2905	39.0
[25]	85,507	36.60	14,916	32.80
[26]	74,600	38.00	15,176	38.00
[28]	<287,160	35.3729	<139,490	38.9982
[33]	1024	30.0	1024	29.0
[34]	5460	48.20	5421	48.20
[36]	200,868	41.20	425,199	35.46
[37]	71,609	48.842	22,709	48.505
[39]	73,231	48.858	23,598	48.553
[44]	73,206	48.868	23,374	48.551
[45]	<71,200	48.6747	<24,965	48.9441
Proposed	196,608	52.3941	196,608	52.4021

**Table 8:** Comparison of the Key space.

Algorithm	[56]	[57]	[58]	[59]	[60]	Proposed
Key Space	2^128^	2^128^	2^199^	2^212^	2^256^	>2^360^

**Table 9:** Speed comparison.

Algorithm	[61]	[62]	[63]	[64]	Proposed
Speed (Mbps)	0.3342	0.3793	0.4080	0.7741	0.93

## Data Availability

Not applicable.

## References

[1] Herman M., Pentek T., Otto B. Design principles for Industrie 4.0 scenarios. Proceedings of the 2016 49th Hawaii International Conference on System Sciences (HICSS).

[2] Thuemmler C., Bai C. (2017). Health 4.0: Application of Industry 4.0: Design Principles in Future Asthma Management. Health 4.0: How Virtualization and Big Data are Revolutionizing Healthcare.

[3] Fatima I., Malik S.U.R., Anjum A., Ahmad N. (2020). Cyber-Physical Systems and IoT: Architectural Practices, Interoperability, and Transformation. IT Prof..

[4] Kim N.Y., Rathore S., Ryu J.H., Park J.H. (2018). A Survey on Cyber-Physical System Security for IoT: Issues, Challenges, Threats, Solutions. J. Inf. Process. Syst..

[5] Al-Garadi M.A., Mohamed A., Al-Ali A.K., Du X., Ali I., Guizani M. (2020). A Survey of Machine and Deep Learning Methods for Internet of Things (IoT) Security. IEEE Commun. Surv. Tutor..

[6] Al-Turjman F., Alturjman S. (2020). 5G/IoT-enabled UAVs for multimedia delivery in industry-oriented applications. Multimed. Tools Appl..

[7] Mohamed N., Al-Jaroodi J. The Impact of Industry 4.0 on Healthcare System Engineering. Proceedings of the 2019 IEEE International Systems Conference (SysCon).

[8] Lopes J.M., Marrone P., Pereira S.L., Dias E.M. (2019). Health 4.0: Challenges for an Orderly and Inclusive Innovation. IEEE Technol. Soc. Mag..

[9] Sudana D., Emanuel A.W.R. How Big Data in Health 4.0 Helps Prevent the Spread of Tuberculosis. Proceedings of the 2019 2nd International Conference on Bioinformatics, Biotechnology and Biomedical Engineering (BioMIC)-Bioinformatics and Biomedical Engineering.

[10] Sayilgan E., İşler Y. Medical devices sector in medical industry 4.0. Proceedings of the 2017 Medical Technologies National Congress (TIPTEKNO).

[11] Parah S.A., Ahad F., Sheikh J.A., Bhat G.M. (2017). Hiding clinical information in medical images: A new high capacity and reversible data hiding technique. J. Biomed. Inform..

[12] Parah S.A., Ahad F., Sheikh J.A., Bhat G.M. (2017). Reversible and high capacity data hiding technique for E-healthcare applications. Multimed. Tools Appl..

[13] Loan N.A., Parah S.A., Sheikh J.A., Akhoon J.A., Bhat G.M. (2017). Hiding Electronic Patient Record (EPR) in medical images: A high capacity and computationally efficient technique for e-health care applications. J. Biomed. Inform..

[14] Parah S.A., Sheikh J.A., Akhoon J.A., Loan N.A., Bhat G.M. (2017). Information hiding in edges: A high capacity information hiding technique using hybrid edge detection. Multimed. Tools Appl..

[15] Parah S.A., Ahad F., Sheikh J.A., Loan N.A., Bhat G.M. (2017). Information Hiding in Medical Images: A Robust Medical Image Watermarking System for E-Healthcare. Multimed. Tools Appl..

[16] Qiu H., Qiu M., Memmi G., Liu M. (2020). Secure Health Data Sharing for Medical Cyber-Physical Systems for the Healthcare 4.0. IEEE J. Biomed. Health Inform..

[17] Jamai I., Ben Azzouz L., Saïdane L.A. Security issues in Industry 4.0. Proceedings of the 2020 International Wireless Communications and Mobile Computing (IWCMC).

[18] Carracedo J.M., Milliken M., Chouhan P.K., Scotney B., Lin Z., Sajjad A., Shackleton M. Cryptography for Security in IoT. Proceedings of the 2018 Fifth International Conference on Internet of Things: Systems, Management, and Security.

[19] Sarmila K.B., Manisekaran S.V. A Study on Security Considerations in IoT Environment and Data Protection Methodologies for Communication in Cloud Computing. Proceedings of the 2019 International Carnahan Conference on Security Technology (ICCST).

[20] Sadhukhan S., Singh M., Majumder K., Chatterjee S., Sarkar S. (2020). A Survey on Security on Medical Data and Images in Healthcare. Proceedings of International Conference on Recent Trends in Machine Learning, IOT Smart Cities and Applications.

[21] Sharma S., Singh S. An Analysis of Reversible Data Hiding Algorithms for Encrypted Domain. Proceedings of the 2019 Third International conference on I-SMAC (IoT in Social, Mobile, Analytics and Cloud) (I-SMAC).

[22] Khari M., Garg A.K., Gandomi A.H., Gupta R., Patan R., Balusamy B. (2020). Securing Data in the Internet of Things (IoT) Using Cryptography and Steganography Techniques. IEEE Trans. Syst. Man Cybern. Syst..

[23] Hamza R., Yan Z., Muhammad K., Bellavista P., Titouna F. (2020). A privacy-preserving cryptosystem for IOT E-healthcare. Inf. Sci..

[24] Hamza R., Hassan A., Patil A.S., Chen X., Huang X., Zhang J. (2019). A Lightweight Secure IoT Surveillance Framework Based on DCT-DFRT Algorithms. Machine Learning for Cyber Security, ML4CS 2019.

[25] Xuan G., Zhu J., Chen J., Shi Y.Q., Ni Z., Su W. (2002). Distortion less data hiding based on integer wavelet transform. IEE Electron. Lett..

[26] Celik M.U., Sharman G., Tekalp A.M., Saber E. Reversible data hiding. Proceedings of the International Conference on Image Processing.

[27] Tian J. (2003). Reversible data embedding using a difference expansion. IEEE Trans. Circuits Syst..

[28] Al-Qershi O.M., Khoo B.E. (2011). High capacity data hiding schemes for medical images based on difference expansion. J. Syst. Softw..

[29] Tseng H.W., Chang C.C. (2008). An extended difference expansion algorithm for reversible watermarking. Image Vis. Comput..

[30] Li X., Zhang W., Gui X., Yang B. (2013). A novel reversible data hiding scheme based on two-dimensional difference-histogram modification. IEEE Trans. Inform. Forensics Secur..

[31] Pan J.S., Yang C.N., Lin C.C., Wang Z.H., Chang C.C., Li M.L. (2013). Multi-dimensional and Multi-level Histogram-Shifting-Imitated Reversible Data Hiding Scheme. Adv. Intell. Syst. Appl..

[32] Tai W., Yeh C., Chang C. (2009). Reversible data hiding based on histogram modification of pixel differences. IEEE Trans. Circuits Syst. Video Technol..

[33] Vleeschouwer C.D., Delaigle J.F., Macq B. Circular interpretation of histogram for reversible watermarking. Proceedings of the 2001 IEEE Fourth Workshop on Multimedia Signal Processing.

[34] Ni Z., Shi Y., Ansari N., Su W. (2006). Reversible data hiding. IEEE Trans. Circuits Syst. Video Technol..

[35] Tsai P., Hu Y.C., Yeh H.L. (2009). Reversible image hiding scheme using predictive coding and histogram shifting. Signal Process..

[36] Jung K., Yoo K. (2009). Data hiding method using image interpolation. Comput. Stand. Interfaces.

[37] Luo L., Chen Z., Chen M., Zeng X., Xiong Z. (2010). Reversible image watermarking using interpolation technique. IEEE Trans. Inf. Forensics Secur..

[38] Abadi M.A.M., Danyali H., Helfroush M.S. Reversible watermarking based on interpolation error histogram shifting. Proceedings of the 5th International Symposium on Telecommunications (IST).

[39] Naheed T., Usman I., Dar A. Lossless data hiding using optimized interpolation error expansion. Proceedings of the 2011 Frontiers of Information Technology.

[40] Lee C.F., Huang Y.L. (2012). An efficient image interpolation increasing payload in reversible data hiding. Expert Syst. Appl..

[41] Jie H., Tianrui L. (2015). Reversible steganography using extended image interpolation technique. Comput. Electr. Eng..

[42] Tang M., Jie H., Wen S. (2014). A high capacity image steganography using multilayer embedding. Optik.

[43] Arsalan M., Sana A.M., Asifullah K. (2012). Intelli-gent reversible watermarking in integer wavelet domain for medical images. J. Syst. Softw..

[44] Naheed T., Imran U., Tariq M.K., Amir H.D., Muhammad F.S. (2014). Intelligent reversible watermarking technique in medical images using GA and PSO. Optik.

[45] Wang X.T., Chang C.C., Nguyen T.S., Li M.C. (2013). Reversible data hiding for high-quality images exploiting interpolation and direction order mechanism. Digit Signal Process..

[46] Wahed M.A., Nyeem H., Elahi M.F. An Improved Interpolation based Reversible Data Hiding for Medical Images. Proceedings of the 2019 International Conference on Electrical, Computer and Communication Engineering (ECCE).

[47] Wahed M.A., Nyeem H. Efficient Data Embedding for Interpolation based Reversible Data Hiding Scheme. Proceedings of the 2017 2nd International Conference on Electrical & Electronic Engineering (ICEEE).

[48] Chang C., Nguyen T., Liu Y. A reversible data hiding scheme for image interpolation based on reference matrix. Proceedings of the 2017 5th International Workshop on Biometrics and Forensics (IWBF).

[49] Mathew T., Johnpaul C.I. Reversible data hiding in encrypted images using interpolation-based distributed space reservation. Proceedings of the 2017 4th International Conference on Advanced Computing and Communication Systems (ICACCS).

[50] Nazari M., Mehrabian M. (2020). A novel chaotic IWT-LSB blind watermarking approach with flexible capacity for secure transmission of authenticated medical images. Multimed. Tools Appl..

[51] Hemdan E.E.D. (2020). An efficient and robust watermarking approach based on single value decompression, multi-level DWT, and wavelet fusion with scrambled medical images. Multimed. Tools Appl..

[52] Garcia-Guerrero I.E.E., Inzunza-Gonzalez E., Lopez-Bonilla O.R., Cardenas-Valdez J.R., Tlelo-Cuautle E. (2020). Randomness improvement of chaotic maps for image encryption in a wireless communication scheme using PIC-microcontroller via zigbee channels. Chaos Solitons Fractals.

[53] Malik M.G.A., Bashir Z., Iqbal N., Imtiaz M.A. (2020). Color Image Encryption Algorithm Based on Hyper-Chaos and DNA Computing. IEEE Access.

[54] Ergün S. Security analysis of a chaos-based image encryption scheme. Proceedings of the 2018 19th IEEE Mediterranean Electrotechnical Conference (MELECON).

[55] Suneja K., Dua S., Dua M. A Review of Chaos-based Image Encryption. Proceedings of the 2019 3rd International Conference on Computing Methodologies and Communication (ICCMC).

[56] Li A.R., Liu Q., Liu L. (2019). Novel image encryption algorithm based on improved logistic map. IET Image Process..

[57] Wang H., Xiao D., Chen X., Huang H. (2018). Cryptanalysis and enhancements of image encryption using combination of the 1D chaotic map. Signal Process..

[58] Chen J., Chen L., Zhang L.Y., Zhu Z.L. (2019). Medical image cipher using hierarchical diffusion and non-sequential encryption. Nonlinear Dyn..

[59] Hanis S., Amutha R. (2019). A fast double-keyed authenticated image encryption scheme using an improved chaotic map and a butter y-like structure. Nonlinear Dyn..

[60] Zhu H., Zhao Y., Song Y. (2019). 2D Logistic-modulated-sine-coupling-Logistic chaotic map for image encryption. IEEE Access.

[61] Ping P., Wu J., Mao Y., Xu F., Fan J. (2018). Design of image cipher using life-like cellular automata and chaotic map. Signal Process..

[62] Luo Y., Zhou R., Liu J., Qiu S., Cao Y. (2018). An efficient and self-adapting colour-image encryption algorithm based on chaos and interactions among multiple layers. Multimed. Tools Appl..

[63] Wang X., Zhu X., Zhang Y. (2018). An image encryption algorithm based on Josephus traversing and mixed chaotic map. IEEE Access.

[64] Wu J., Liao X., Yang B. (2018). Cryptanalysis and enhancements of image encryption based on three-dimensional bit matrix permutation. Signal Process..

[65] Jia H., Ren H., Bai C., Li J. Hyper-chaos encryption application in intelligent home system. Proceedings of the International Conference On Smart Technologies for Smart Nation (Smart Tech Con).

[66] Zhan K., Wei D., Shi J., Yu J. (2017). Cross-utilizing hyperchaotic and DNA sequences for image encryption. J. Electron. Imaging.

[67] Zhang X., Wang L., Wang Y., Niu Y., Li Y. (2020). An Image Encryption Algorithm Based on Hyperchaotic System and Variable-Step Josephus Problem. Int. J. Opt..

[68] Tong X., Liu Y., Zhang M., Xu H., Wang Z. (2015). An Image Encryption Scheme Based on Hyperchaotic Rabinovich and Exponential Chaos Maps. Entropy.

